# pSynGAP1 disturbance-mediated hippocampal oscillation network impairment might contribute to long-term neurobehavioral abnormities in sepsis survivors

**DOI:** 10.18632/aging.104080

**Published:** 2020-11-16

**Authors:** Yong Wang, Hua Wei, Jianhua Tong, Muhuo Ji, Jianjun Yang

**Affiliations:** 1 Department of Anesthesiology, Pain and Perioperative Medicine, The First Affiliated Hospital of Zhengzhou University, Zhengzhou, China

**Keywords:** proteomic, SynGAP, oscillation, cognitive dysfunction

## Abstract

Although more patients survive sepsis and are increasingly discharged from the hospital, they often experience long-term cognitive and psychological impairment with significant socioeconomic impact. However, the pathophysiological mechanisms have not been fully elucidated. In the present study, we showed that LPS induced long-term neurobehavioral abnormities, as reflected by significantly decreased freezing time to context and sucrose preference. Using a high-throughput quantitative proteomic screen, we showed that phosphorylation of synaptic GTPase-activating protein 1 (pSynGAP1) was identified as the hub of synaptic plasticity and was significantly decreased following LPS exposure. This decreased pSynGAP was associated with significantly lower theta and gamma oscillations in the CA1 of the hippocampus. Notably, restoration of pSynGAP1 by roscovitine was able to reverse most of these abnormities. Taken together, our study suggested that pSynGAP1 disturbance-mediated hippocampal oscillation network impairment might play a critical role in long-term neurobehavioral abnormities of sepsis survivors.

## INTRODUCTION

Sepsis, a disease with a high and increased prevalence worldwide, is the major cause of critical illness resulting in admission to Intensive Care Unit. Although more patients survive sepsis and are increasingly discharged from the hospital, they often experience long-term cognitive and psychological impairment with significant socioeconomic impact [1–3]. Previous studies have demonstrated neurobehavioral abnormities after sepsis by lipopolysaccharide (LPS) challenge or cecal ligation and puncture (CLP) in rodent models of sepsis [4–6]. However, the reported studies primarily focused on the relatively short stage after sepsis development. Given the possibility that infections in early life may be associated with increased risk of Alzheimer’s Disease (AD) [7], understanding the long-term impact of sepsis on brain function and its pathophysiological mechanisms are urgently needed.

Proteomic approach has grown rapidly and is a powerful and promising tool in identifying disease phenotypes, drug targets, and clinical biomarkers [8]. With the development of proteomic techniques, such as isobaric tagging for relative and absolute quantitation (iTRAQ) with liquid chromatography-mass spectrometry (LC-MS) analyses, have greatly improved the detection ability and reproducibility. It has been widely used in exploring the molecular markers and mechanisms in various diseases, including cancer, cardiovascular diseases, and psychiatric illnesses [9–12]. Although various mechanisms that contribute to the pathogenesis of sepsis-induced neurobehavioral abnormities have been revealed by studies on individual genes or proteins, systematic analysis of the hippocampal proteomic profile is still lacking.

Therefore, the aim of the present study was to investigate the long-term neurobehavioral alterations one year after CLP or LPS challenge. Our study showed that LPS challenge but not CLP induced long-term neurobehavioral abnormities in sepsis survivors, we thus focused our research on LPS-induced animals. Given the key role of hippocampal oscillation network in cognitive function, we tested whether hippocampal oscillations would be affected by LPS exposure, and if so, whether that effect would be mediated by impaired synaptic plasticity.

## RESULTS

### Survival rate

**Figure 1:**
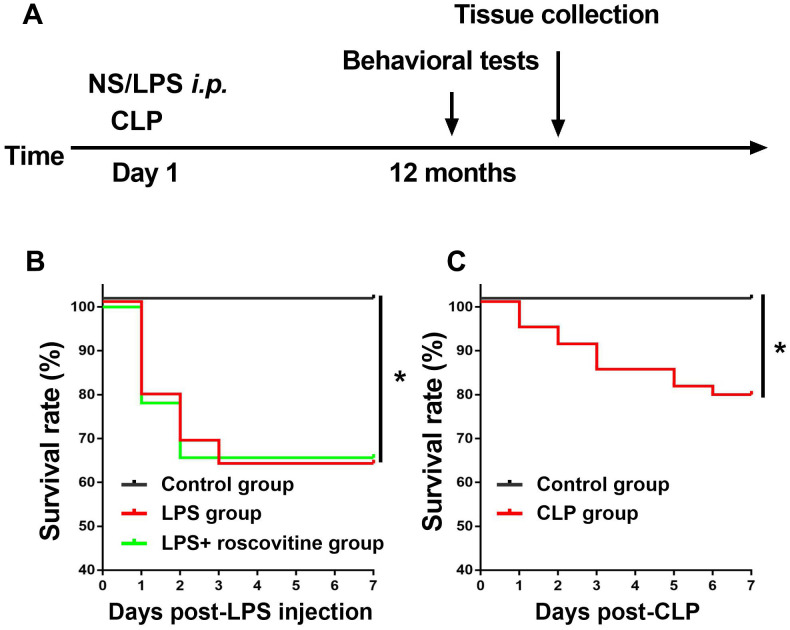
(**A**) Timeline of the experimental procedures of the present study. (**B**) Effects of LPS on survival rate, n = 25 for control group, n = 40 for LPS group, and n=33 for LPS + roscovitine group. (**C**) Effects of CLP on survival rate, n = 22 for control group, n = 52 for CLP group. LPS, lipopolysaccharide; NS, normal saline, *P < 0.05.

We observed 7-day survival rate after LPS injection or CLP, we showed the survival rate was 63% in the LPS group and 66% in the LPS + roscovitine group, which was significantly lower than that in the control group (P = 0.0066, Figure 1B). In the current study, many animals died during the observation period. Ultimately, 21 mice in the control group, 20 mice in the LPS group, and 18 mice in the LPS + roscovitine group survived before behavior tests. In addition, CLP induced significantly decreased survival rate (78.846%) compared with sham group (100%) (P = 0.0259, Figure 1C).

### Identification of altered proteins in the hippocampus of LPS-exposed mice

**Figure 2:**
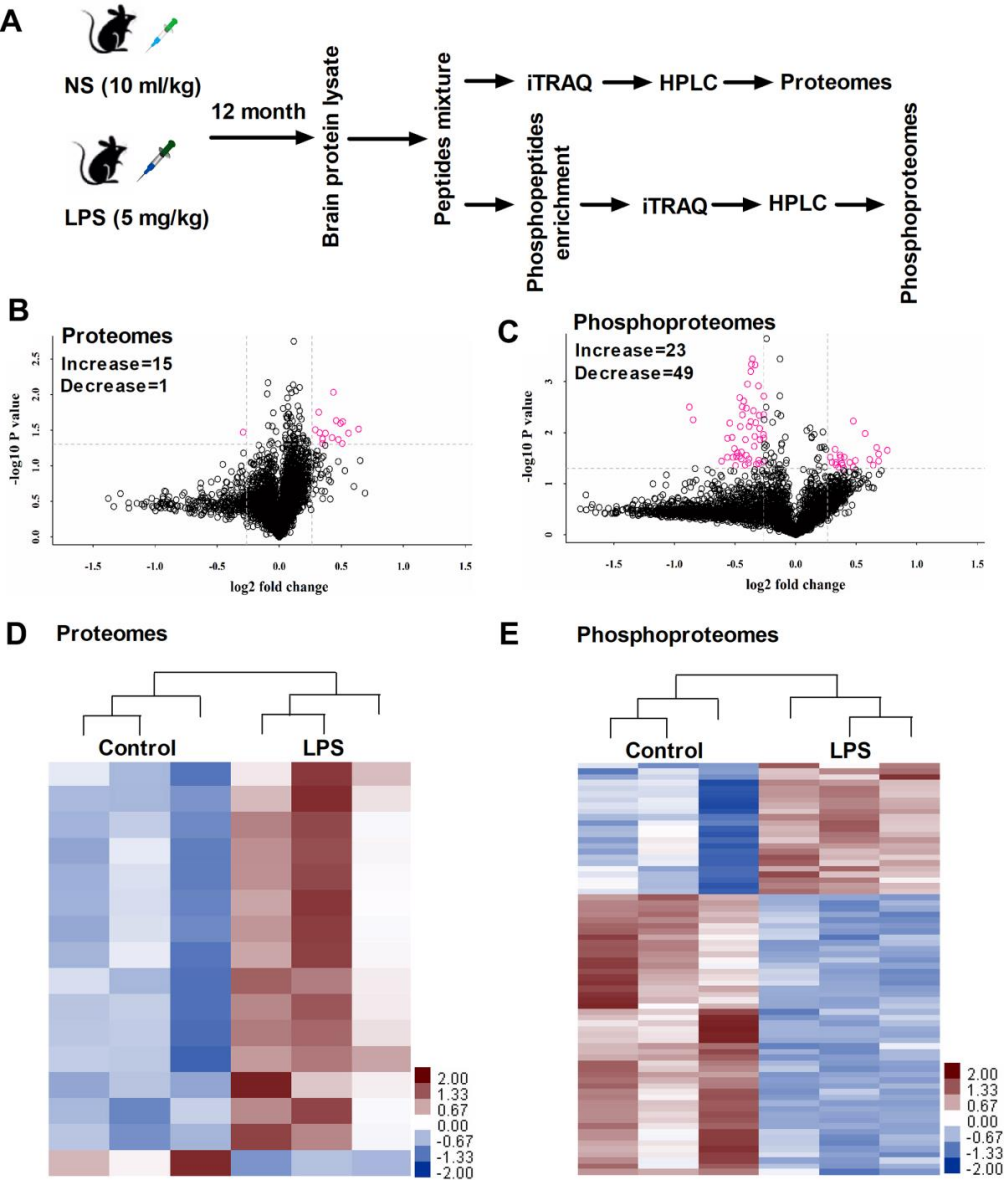
(**A**) Timeline of the proteins and phosphoproteins procedure. (**B, C**) Volcano plot indicating significantly altered proteins and phosphoproteins between control and LPS groups. (**D, E**) Heat map showing significantly altered proteins and phosphoproteins between control and LPS groups (n = 3). LPS, lipopolysaccharide; NS, normal saline; ITRAQ, isobaric tagging for relative and absolute quantitation; HPLC, High performance liquid chromatography.

Figure 2A shows the design for proteomic and phosphoproteomic analysis. We used the iTRAQ approach and performed large-scale quantitative analysis. We identified 25045 unique peptides and 4163 proteins from each sample. Significant differences in protein expressions were determined by the following threshold: “*P* ≤ 0.05 and fold change ≥ 1.2 or <0.83”, which has been adopted by previous study [9]. Accordingly, 16 proteins showing significant changes were observed in hippocampal samples between control and LPS-exposed mice (Figure 2B and Table 1). Briefly, Rars2, Clic6, Rttn, Gtpbp10, Itgb7, Hbb-bh0, Sept10, Mmp1a, Eml1, Krt8, Ubqln4, Serpini1, Ca5b, Elmo1, Gng10 were significantly upregulated, while Smad1 was downregulated. These differentially expressed proteins were displayed using a heatmap (Figure 2D). Gene ontology (GO) classification showed these differentially expressed proteins involved in different molecular function categories, and participated in many biological processes (Figure 4). The GO data provided an overview showing that abundant changes are exhibited by changes in structural protein, extracellular matrix, membrane protein composition, and enzyme catalytic activities following LPS challenge.

**Table 1:** Differential proteomics in hippocampus by iTRAQ analysis from control and LPS mice.

**Accession numbers**	**Protein names**	**Gene names**	**Unique peptides**	**Peptides coverage**	**Fold change**
**Q3U186**	Rars2	Rars2	1	2.7681661	1.55797
**Q8BHB9**	Clic6	Clic6	1	2.1812081	1.47219
**Q8R4Y8**	Rttn	Rttn	2	0.7637017	1.42522
**Q8K013**	Gtpbp10	Gtpbp10	1	6.010929	1.42171
**P26011**	Itgb7	Itgb7	1	1.1166253	1.4073
**P04443**	Hbb-bh0	Hbb-bh0	1	6.8027211	1.39139
**Q8C650**	Sept10	Sept10	1	3.7610619	1.37436
**Q9EPL5**	Mmp1a	Mmp1a	1	3.6637931	1.35294
**Q05BC3**	Eml1	Eml1	1	1.3513514	1.34336
**P11679**	Krt8	Krt8	3	8.9795918	1.29396
**Q99NB8**	Ubqln4	Ubqln4	1	7.7181208	1.27273
**O35684**	Serpini1	Serpini1	1	5.6097561	1.27273
**Q9QZA0**	Ca5b	Ca5b	1	5.362776	1.2531
**Q8BPU7**	Elmo1	Elmo1	2	9.3535076	1.24682
**Q9CXP8**	Gng10	Gng10	1	22.058824	1.22305
**P70340**	Smad1	Smad1	1	3.0107527	0.81683

We further screened the phosphoproteins based on their fold-changes in expression level. We identified 72 proteins that showed at least a 20% increase or decrease in expression, of which 23 were upregulated and 49 were downregulated (Figure 2C and Table 2). These differentially expressed proteins were also displayed using a heatmap (Figure 2E). We noted a significantly increased expression of Cdk11b, indicating that the neurons could undergo apoptosis following LPS challenge. Also, LPS also increased microtubule-associated proteins, such as Map1b, Dctn1, Map2, and Ank2 relative to controls. In addition to neuronal cell signaling pathway, we have identified several biological functions altered in LPS-exposed mice, such as Syngap1, Bsn, Shisa6, Synpo, Pclo, Ppp1r9b, and Dlgap2 for synaptic related proteins, suggesting a potential mechanistic link between the synaptic dysfunction and LPS-induced neurobehavioral abnormities.

**Table 2:** Differential phosphoproteomics in hippocampus by iTRAQ analysis from control and LPS mice.

**Accession numbers**	**Gene names**	**Description**	**Fold change**
P24788	Cdk11b	Cyclin-dependent kinase 11B	1.68937696
Q9QYR6	Map1a	Microtubule-associated protein 1A	1.60799652
P14869	Rplp0	60S acidic ribosomal protein P0	1.60799652
P14873	Map1b	Microtubule-associated protein 1B	1.58955546
P47955	Rplp1	60S acidic ribosomal protein P1	1.55754476
Q9QYR6	Map1a	Microtubule-associated protein 1A	1.52737995
P70704	Atp8a1	Phospholipid-transporting ATPase IA	1.48797678
O08788	Dctn1	Dynactin subunit 1	1.40730337
Q91YM2	Arhgap35	Rho GTPase-activating protein 35	1.39043825
P20357	Map2	Microtubule-associated protein 2	1.38893312
Q9QWY8	Asap1	Arf-GAP with SH3 domain, ANK repeat and PH domain-containing protein 1	1.36127509
A2ARP1	Ppip5k1	Inositol hexakisphosphate and diphosphoinositol-pentakisphosphate kinase 1	1.31979892
Q9JIS5	Sv2a	Synaptic vesicle glycoprotein 2A	1.31570822
Q80YE4	Aatk	Serine/threonine-protein kinase LMTK1	1.30680507
Q4QQM5	Miga1	Mitoguardin 1	1.30237913
O54781	Srpk2	SRSF protein kinase 2	1.29231945
Q9D7P6	Iscu	Iron-sulfur cluster assembly enzyme ISCU, mitochondrial	1.28783835
Q8C8R3	Ank2	Ankyrin-2	1.27703985
Q6PB44	Ptpn23	Tyrosine-protein phosphatase non-receptor type 23	1.25856229
Q9JMH9	Myo18a	Unconventional myosin-XVIIIa	1.25818592
Q8BP99		UPF0500 protein C1orf216 homolog	1.25309801
Q80TL4	Phf24	PHD finger protein 24	1.23094087
Q8CC27	Cacnb2	Voltage-dependent L-type calcium channel subunit beta-2	1.21856509
P56399	Usp5	Ubiquitin carboxyl-terminal hydrolase 5	0.83323152
Q9Z2H5	Epb41l1	Band 4.1-like protein 1	0.83318057
Q3UHJ0	Aak1	AP2-associated protein kinase 1	0.83262065
Q01815	Cacna1c	Voltage-dependent L-type calcium channel subunit alpha-1C	0.83150183
P14873	Map1b	Microtubule-associated protein 1B	0.83119658
Q9WTX2	Prkra	Interferon-inducible double-stranded RNA-dependent protein kinase activator A	0.81518451
Q8C8R3	Ank2	Ankyrin-2	0.81378476
Q9EPJ9	Arfgap1	ADP-ribosylation factor GTPase-activating protein 1	0.8115942
Q99JX3	Gorasp2	Golgi reassembly-stacking protein 2	0.80965309
Q80TI0	Gramd1b	GRAM domain-containing protein 1B	0.80965309
Q3V3V9	Carmil2	Capping protein, Arp2/3 and myosin-I linker protein 2	0.80856195
Q9Z1B3	Plcb1	1-phosphatidylinositol 4,5-bisphosphate phosphodiesterase beta-1	0.79802218
Q9Z2H5	Epb41l1	Band 4.1-like protein 1	0.79533214
Q9QWI6	Srcin1	SRC kinase signaling inhibitor 1	0.79265013
F6SEU4	Syngap1	Ras/Rap GTPase-activating protein SynGAP	0.79104478
Q3TY60	Fam131b	Protein FAM131B	0.78890877
Q7TME0	Plppr4	Phospholipid phosphatase-related protein type 4	0.78518298
O88737	Bsn	Protein bassoon	0.78071217
Q9JM52	Mink1	Misshapen-like kinase 1	0.77619893
G3XA57	Rab11fip2	Rab11 family-interacting protein 2	0.77327816
D3YVF0	Akap5	A-kinase anchor protein 5	0.77304965
Q61097	Ksr1	Kinase suppressor of Ras 1	0.76626435
P20357	Map2	Microtubule-associated protein 2	0.765
Q3UHD9	Agap2	Arf-GAP with GTPase, ANK repeat and PH domain-containing protein 2	0.76315016
Q68EF6	Begain	Brain-enriched guanylate kinase-associated protein	0.75901495
P33173	Kif1a	Kinesin-like protein KIF1A	0.75416545
Q9R0K7	Atp2b2	Plasma membrane calcium-transporting ATPase 2	0.75416545
Q9WV92	Epb41l3	Band 4.1-like protein 3	0.75
Q924A2	Cic	Protein capicua homolog	0.74876129
Q3UH99	Shisa6	Protein shisa-6	0.73690304
O54829	Rgs7	Regulator of G-protein signaling 7	0.73589818
P48453	Ppp3cb	Serine/threonine-protein phosphatase 2B catalytic subunit beta isoform	0.73260179
P97427	Crmp1	Dihydropyrimidinase-related protein 1	0.7323903
Q9Z0P4	Palm	Paralemmin-1	0.72860847
Q8K2Y9	Ccm2	Cerebral cavernous malformations protein 2 homolog	0.72612198
Q9QYG0	Ndrg2	Protein NDRG2	0.72562554
Q8CC35	Synpo	Synaptopodin	0.71604232
Q9QYX7	Pclo	Protein piccolo	0.71253212
Q3UHD9	Agap2	Arf-GAP with GTPase, ANK repeat and PH domain-containing protein 2	0.70842825
P04370	Mbp	Myelin basic protein	0.70426136
Q9CYZ2	Tpd52l2	Tumor protein D54	0.69875425
P35803	Gpm6b	Neuronal membrane glycoprotein M6-b	0.69491525
O88703	Hcn2	Potassium/sodium hyperpolarization-activated cyclic nucleotide-gated channel 2	0.68586682
Q6R891	Ppp1r9b	Neurabin-2	0.67691448
Q8BJ42	Dlgap2	Disks large-associated protein 2	0.67597765
Q80TJ1	Cadps	Calcium-dependent secretion activator 1	0.65425972
O55131	Sept7	Septin-7	0.5560166
Q5FWK3	Arhgap1	Rho GTPase-activating protein 1	0.54400412

**Figure 3:**
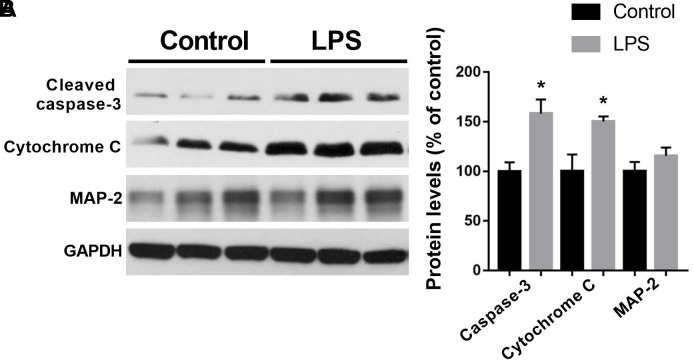
**Expressions of cleaved caspase-3, cytochrome C, and MAP-2 in the hippocampus by western blotting analysis.** (**A**) Representative Western blots bands of cleaved caspase-3, cytochrome C, and MAP-2 in the hippocampus; (**B**) Quantitative analysis of cleaved caspase-3, cytochrome C, and MAP-2. Data are presented as the mean ± SEM, n = 4, *P < 0.05.

Next, we performed some of the functional changes with regarding to apoptosis, mitochondria dysfunction, and microtubule formation. As shown in Figure 3, we showed that LPS induced significantly increased expressions of cleaved caspase-3 (t = 3.413, *P* = 0.0143) and cytochrome C (t = 2.844, *P* = 0.0294) in the hippocampus as compared with the control group, two markers of apoptosis and mitochondria dysfunction, respectively. However, there was no difference in microtubule-associated protein such as microtubule-associated protein-2 (MAP-2) between the control and LPS groups (t = 1.241, *P* = 0.2609).

**Figure 4:**
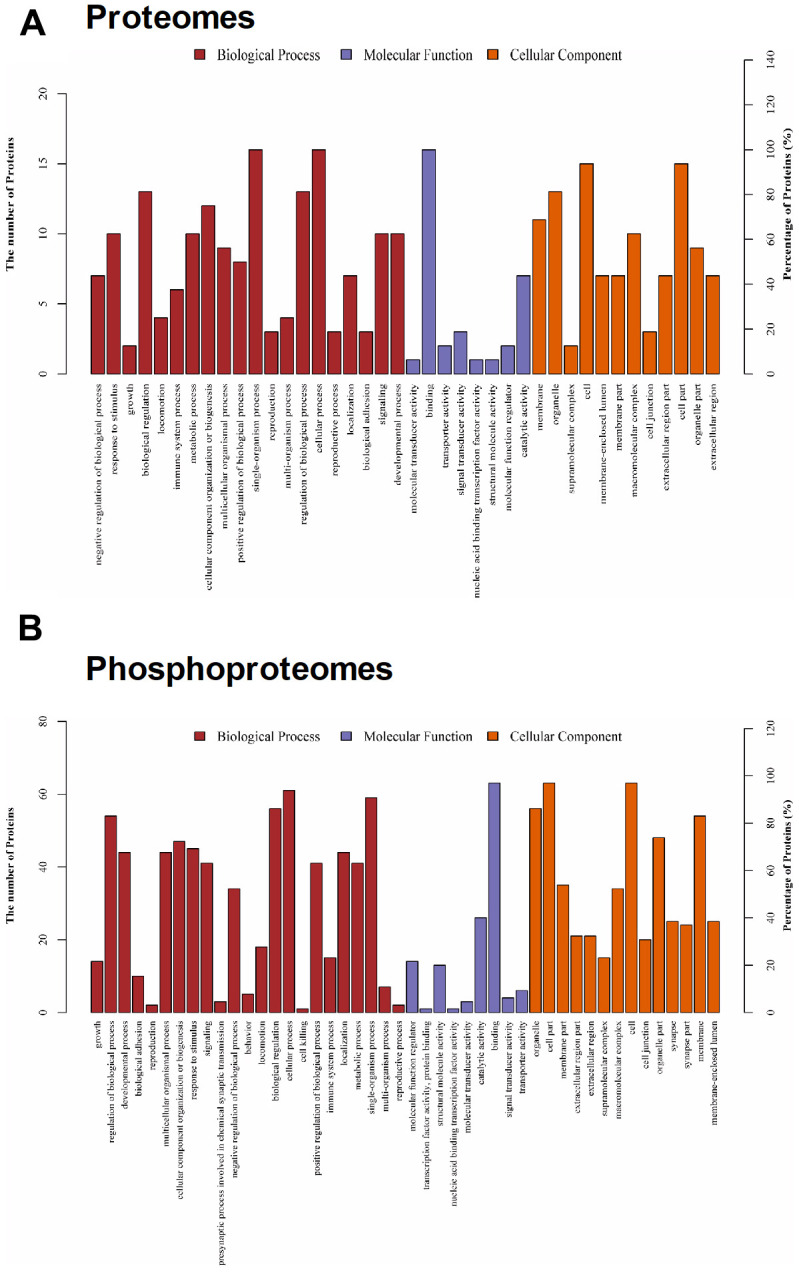
**Gene ontology (GO) classification for differentially expressed proteins.** (**A**) For proteomes, populations of proteins that showed alteredexpression are indicated based on their GO for molecular function, biological process, and cellular components. (**B**) For phosphoproteomes, populations of proteins that showed alteredexpression are indicated based on their GO for molecular function, biological process, and cellular components.

### LPS induced significantly decreased pSynGAP1

**Figure 5:**
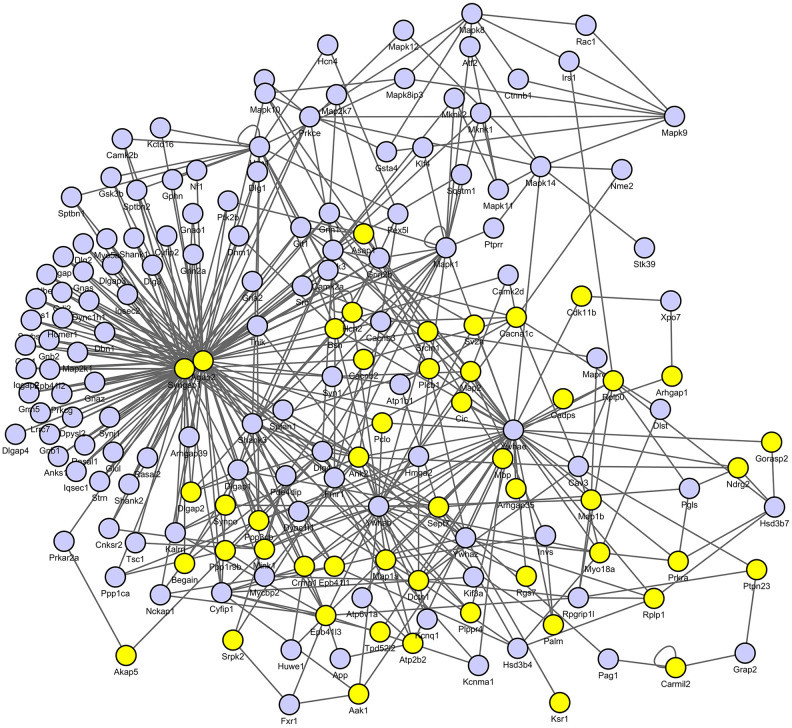
**Protein–protein interaction (PPI) networks of differential proteins between control and LPS groups.** The PPI analysis was based on fold change of protein–protein interaction, which showed SynGAP1 was identified as the hub of synaptic plasticity.

**Figure 6:**
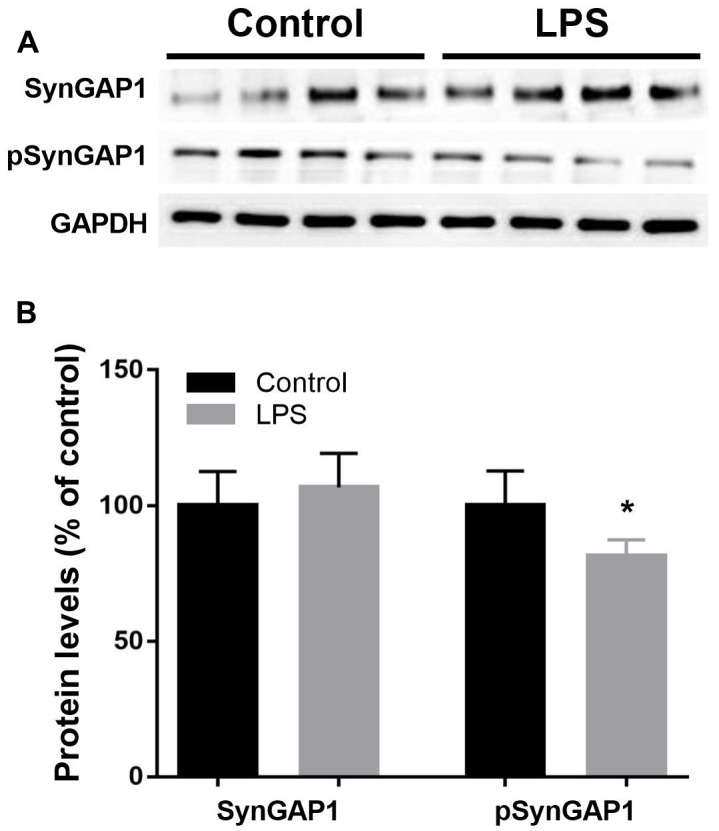
**Validation of Syngap1 and pSyngap1 in hippocampus by western blotting analysis.** (**A**) Representative Western blots bands of Syngap1 and pSyngap1 in the hippocampus; (**B**) Quantitative analysis of Syngap1 and pSyngap1 levels between groups. Data are presented as the mean ± SEM, n = 4, *P < 0.05.

Next, we constructed pathological protein–protein interaction (PPI) networks based on significantly changed phosphoproteins. As shown in Figure 5, nodes indicate proteins with increased or decreased phosphorylation and the proteins directly connected to the altered proteins in the PPI database (blue). In particular, we detected SynGAP1 is among the hub of synaptic plasticity. To further verify the reliability of the iTRAQ results, we examined SynGAP1 and pSynGAP1 levels using the western blot approach (Figure 6). Although SynGAP1 was not affected (t = 0.7615, *P* = 0.4725), we showed that pSynGAP1 in the hippocampus was significantly decreased in LPS-exposed mice (t = 2.632, *P* = 0.039), suggesting the iTRAQ results in the present study were reliable.

### Decreased hippocampal pCamKII, pSynGAP levels, and dendritic spine density following LPS challenge were rescued by roscovitine

**Figure 7:**
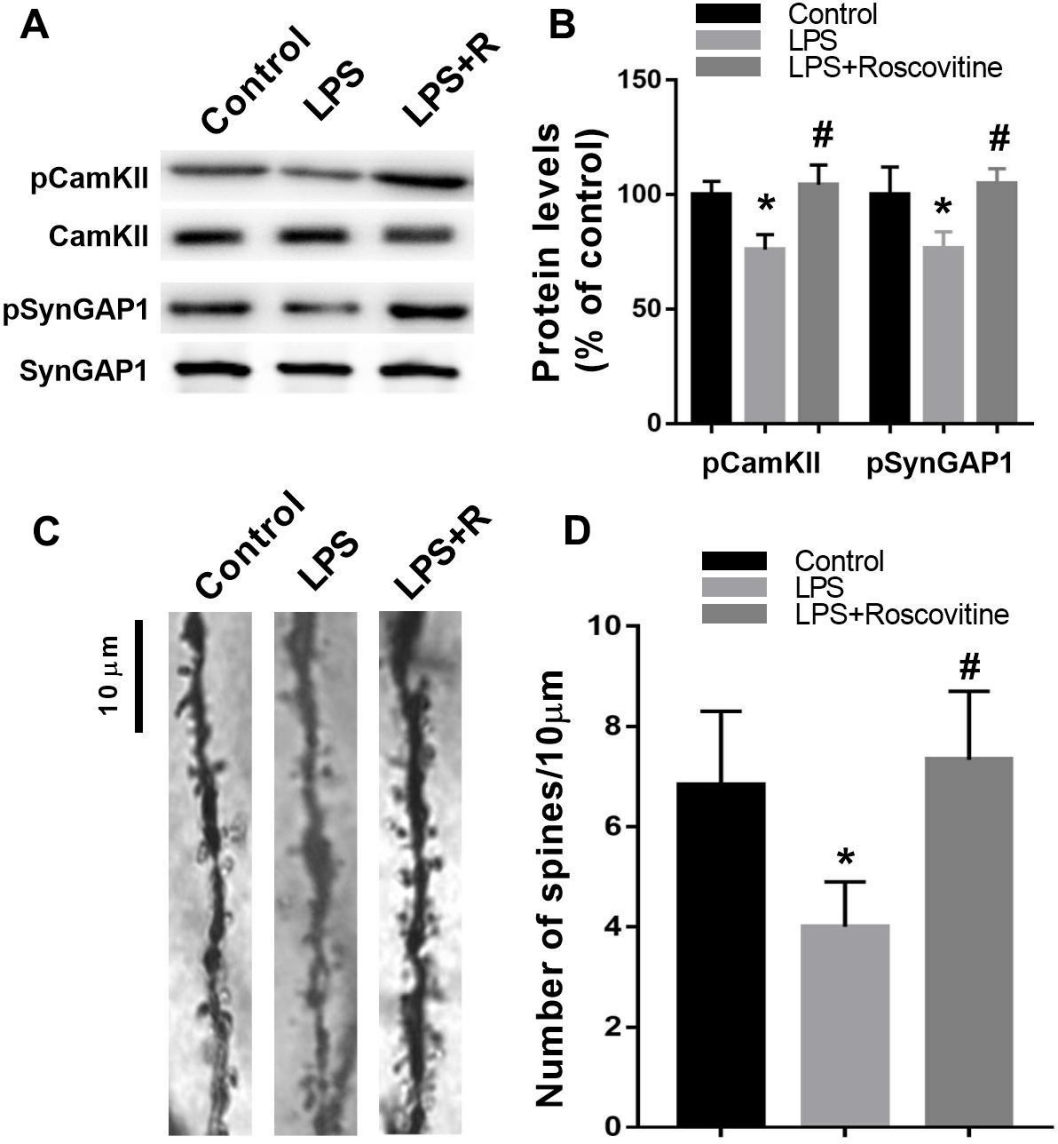
**Decreased hippocampal pCamKII, pSynGAP levels, and dendritic spine density following LPS challenge were rescued by roscovitine.** (**A**, **B**) LPS induced significantly decreased hippocampal pCamKII and pSynGAP levels, which were prevented by roscovitine treatment. (**C**, **D**) LPS induced significantly increased hippocampal dendritic spine loss, which was reversed by roscovitine treatment. Data are presented as the mean ± SEM, n = 4, *P < 0.05 vs control group; #*P* < 0.05 vs LPS group. LPS, lipopolysaccharide.

It has been suggested that Cdk5 inhibition increased pCamKII, which further can increase pSynGAP levels [13]. For this reason, we determined whether Cdk5 inhibition by roscovitine can increase hippocampal pCamKII and pSynGAP levels. As shown in Figure 7A, 7B, roscovitine administration increased hippocampal pCamKII (F_2,9_ = 18.56, *P* = 0.0007) and pSynGAP (F_2,9_ = 11.19, *P* = 0.0035) levels in LPS + roscovitine group compared with LPS group. In addition, LPS significantly reduced dendritic spine density, while roscovitine administration attenuated LPS-induced dendritic spine loss (F_2,9_ = 6.182, *P* = 0.011; Figure 7C, 7D).

### Decreased theta and gamma oscillations in the CA1 of the hippocampus following LPS challenge were prevented by roscovitine

**Figure 8:**
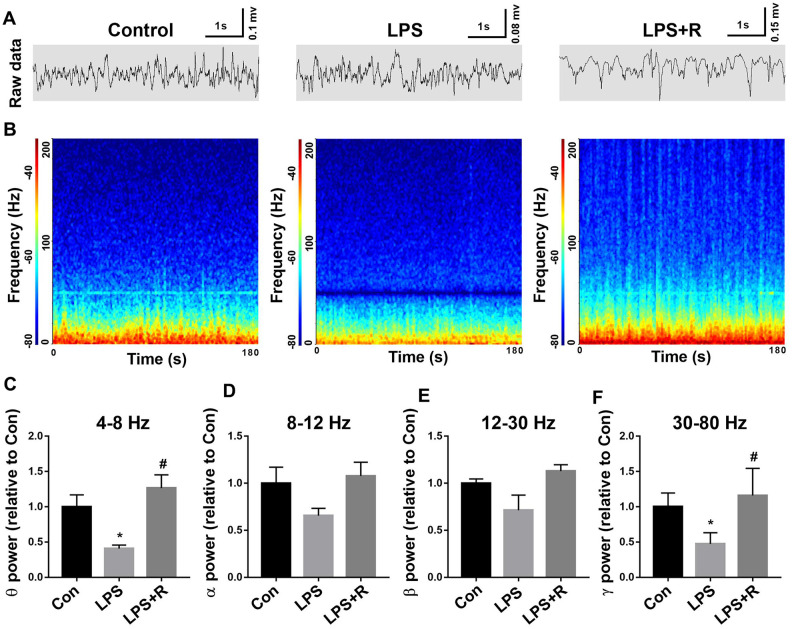
**Decreased gamma oscillation in the CA1 of the hippocampus following LPS challenge was prevented by roscovitine.** (**A**, **B**) Example recordings and example power spectra in the hippocampal. (**C**–**F**) Summary of LFP power, including θ, α, β, and γ oscillation. The theta and gamma oscillation powers were significantly lower in LPS group when compared with control group, which were prevented by roscovitine. Data are shown as mean ± SEM, n = 4, *P < 0.05 vs control group; #*P* < 0.05 vs LPS group. LPS, lipopolysaccharide; R, roscovitine.

It has been shown that Syngap1 plays a critical role in network function [14], we further examined whether decreased pSynGAP1 affected brain oscillations in the CA1 of the hippocampus following LPS challenge. As shown in Figure 8, we found that theta and gamma oscillation power were significantly reduced in the LPS group, which were rescued by roscovitine (theta oscillation: F_2,9_ = 8.799, *P* = 0.0076; gamma oscillation: F_2,9_ = 7.391, *P* = 0.0126). However, there was no difference in α and β oscillation power among groups (alpha oscillation: F_2,9_ = 2.697, *P* = 0.1209; beta oscillation: F_2,9_ = 4.13, *P* = 0.0534).

### LPS-induced neurobehavioral abnormities were attenuated by roscovitine

**Figure 9:**
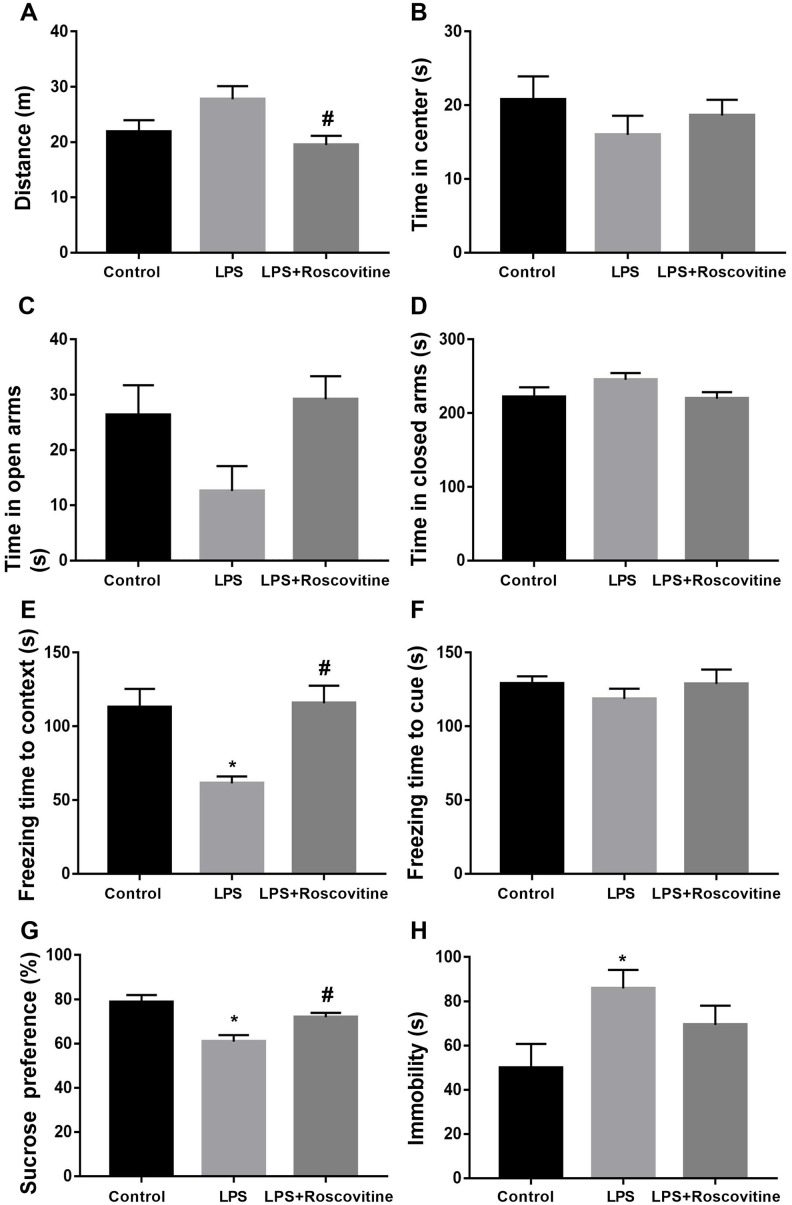
**LPS-induced neurobehavioral abnormities were attenuated by roscovitine.** (**A**) LPS had no effect on the total distance traveled, while roscovitine treatment significantly increased total distance traveled in LPS + roscovitine group compared with LPS group. (**B**) No difference in time spent in the center of the open arena was observed among groups. (**C**, **D**) There was no difference in time in the open arms and closed arms between groups. (**E**) LPS-induced significantly decreased the freezing time to context was reversed by roscovitine treatment. (**F**) There was no difference in freezing time to tone in the auditory-cued fear test among groups. (**G**) Decreased preference for sucrose in LPS-exposed mice was reversed by roscovitine treatment. (**H**) LPS significantly increased immobility compared with control group, which was not prevented by roscovitine treatment. Data are presented as the mean ± SEM, n = 10-12, *P < 0.05 vs control group; #*P* < 0.05 vs LPS group. LPS, lipopolysaccharide.

**Figure 10:**
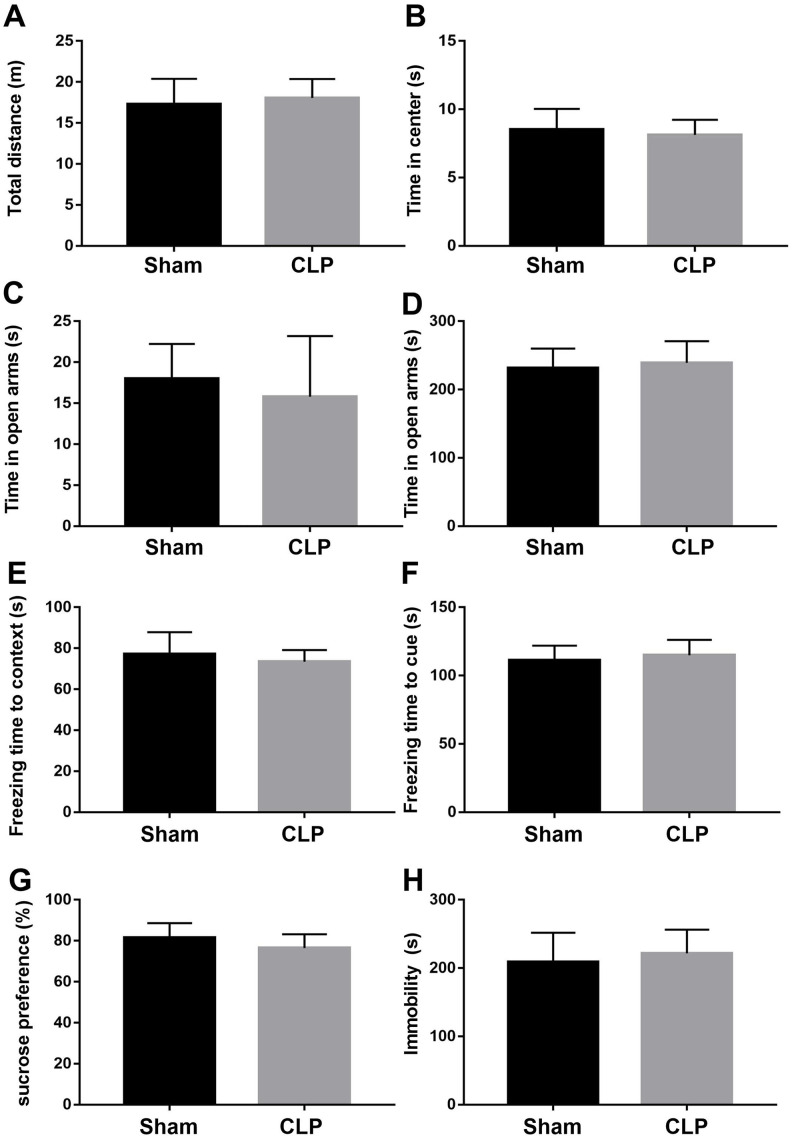
**CLP did not induce neurobehavioral abnormities.** (**A**, **B**) CLP had no effect on the total distance traveled and time spent in the center of the open arena compared with sham group. (**C**, **D**) There was no difference in time in the open arms and closed arms between groups. (**E**, **F**) There was no difference in freezing time to context or tone in fear conditioning tests between groups. (**G**, **H**) CLP had no effect on preference for sucrose or immobility compared with sham group. Data are presented as the mean ± SEM, n = 10-12, *P < 0.05 vs sham group. CLP, cecal ligation and puncture.

The open field test was performed to investigate whether LPS influences locomotor activity and anxiety-like behavior. As shown in Figure 9A, 9B, LPS had no effect on the total distance traveled. However, roscovitine treatment significantly increased total distance traveled in the LPS + roscovitine group compared with LPS group (F_2,31_ = 4.054, *P* = 0.0273). There was no difference in time spent in the center of the open arena among groups (F_2,31_ = 0.7745, *P* = 0.4696). In the elevated plus maze, there was a trend toward a decreased time in the open arms (F_2,31_ = 3.264, *P* = 0.0517, Figure 9C). In addition, no difference in time in the closed arms was observed between groups (F_2,31_ = 1.593, *P* = 0.2194, Figure 9D). However, CLP did not significantly affect total distance traveled (t = 0.1962, *P* = 0.8462, Figure 10A) or time spent in the center (t = 0.2069, *P* = 0.838, Figure 10B) when compared with the sham group. In addition, there was no difference in the time in the open (t = 0.2585, *P* = 0.7985, Figure 10C) or closed arms (t = 0.6287, *P* = 0.536, Figure 10D) between the two groups.

We performed the fear conditioning tests to evaluate whether LPS challenge impaired contextual fear memory. As revealed in Figure 9E, 9F, LPS significantly decreased the freezing time to context relative to that of control group (F_2,31_ = 7.384, *P* = 0.0024), which was reversed by roscovitine treatment. However, there was no difference in freezing time to tone in the auditory-cued fear test among groups (F_2,31_ = 0.561, *P* = 0.5763). Also, there was no difference in freezing time to context (t = 0.3019, *P* = 0.7655, Figure 10E) or tone (t = 0.2329, *P* = 0.818, Figure 10F) between the sham and CLP groups in the fear conditioning tests.

In the sucrose preference test, LPS-exposed mice displayed significantly decreased preference for sucrose relative to that of control group (F2,31 = 10.08, P = 0.0004, Figure 9G). In the forced swim test, LPS significantly increased immobility compared with control group (F2,31 = 3.46, P = 0.044, Figure 9H), suggesting LPS induced depression like behavior. However, roscovitine treatment only reversed the sucrose preference but not immobility. Again, CLP had no effect on the preference for sucrose (t = 1.778, P = 0.0893, Figure 10G) or immobility (t = 1.195, P = 0.2477, Figure 10H) in the CLP group compared with the sham group.

## DISCUSSION

The long-term consequences of sepsis and its pathophysiological mechanisms are complex, and have not been fully elucidated. The large-scale proteomic analysis provides comprehensive information about the regulation of inflammation-associated proteins after sepsis. To the best of our knowledge, this is the first study investigating the long-term neurobehavioral abnormities following LPS exposure. More importantly, we showed that pSynGAP1 disturbance plays a key role in hippocampal oscillation network impairment, which might contribute to long-term neurobehavioral abnormities in sepsis survivors.

Systemic inflammation can impair cognition with relevance to dementia, implying that these acute events induce or exacerbate central nervous system (CNS) pathology, even in the absence of overt invasion of bacteria into the CNS [15]. In animal models of sepsis, induction of neuroinflammation by LPS or CLP increased intracellular accumulation of amyloid precursor protein and amyloid β peptide and consequent cognitive impairments [16, 17]. In human studies, it has been demonstrated that patients with delirium due to sepsis in the Intensive Care Unit showed significant cognitive impairments at 12-18 months after hospital discharge when compared with controls [1]. Recent epidemiological study reports that human survivors of sepsis have an increased risk of long-term cognitive decline such as AD [18]. These classical pathology hallmarks are accompanied by neuroinflammation, synaptic loss, and brain atrophy [4, 5, 19]. Although increasing evidence has suggested that CLP is more clinically relevant, our study showed that LPS challenge but not CLP led to long-term neurobehavioral abnormities. This can be explained by the reason that our model of CLP is mild and thus does not significantly affect brain function. Yet, the mechanism underlying LPS-induced long-term neurobehavioral abnormities remains to be elucidated.

Transcriptomic analysis provided information about the regulation of mRNAs in animal models with sepsis [20]. However, the findings from transcriptomic studies do not always translate into proteome alterations due to post-transcriptional and post-translational regulation mechanisms. The development of quantitative proteomics approaches has greatly accelerated the understanding of various cellular and physiological processes and how these are affected by disease allowing the identification of novel biomarkers. Developments in LC-MS–based proteomics and phosphoproteomics in particular, enable the comprehensive characterization of proteomes and tens of thousands of phosphorylation events [11, 21]. It is a powerful tool for identifying novel molecule biomarkers and also provides insights into the pathophysiology of neurodegenerative and other brain-related diseases [22]. Protein functions can be switched on or off by site-specific phosphorylation, or modulated by cumulative phosphorylation of multiple sites, which is an important posttranslational modification that regulates protein function and plays a prominent role in diverse biological phenomena [23]. Thus, this approach becomes an efficient tool to investigate global signaling-level changes in biological systems [24]. It is estimated that one-third of all proteins are likely to be phosphorylated, thus phosphoproteomic analysis offers an excellent potential for the identification of candidate regulatory proteins in various cellular states. To our knowledge, however, no previous study has utilized this method to investigate long-term hippocampal phosphoproteomic alterations following sepsis development. In the present study, we showed that several cellular signaling cascades related to apoptosis, mitochondria dysfunction, and microtubule-associated protein were significantly up-regulated following LPS, including increased phosphorylation of Cdk11b, Atp8a1, Iscu, Map1b, Dctn1, Map2, and Ank2. Thus, we further performed some of these functional changes and showed that LPS exposure induced apoptosis and mitochondria dysfunction, as reflected by significantly increased cleaved caspase-3 and cytochrome C expressions in the hippocampus. These results are consistent with previous findings in the literature indicating that apoptosis, mitochondria dysfunction, and disturbance in microtubule-associated protein are required for hippocampus-dependent learning [25–27]. However, LPS did not significantly affect microtubule-associated protein such as MAP-2, suggesting microtubule-associated protein dysfunction may be not an important factor contributing to long-term neurobehavioral abnormities induced by LPS. In addition to neuronal cell signaling pathway, we have also identified several biological functions altered in LPS-exposed mice, such as Syngap1, Bsn, Shisa6, Synpo, Pclo, Ppp1r9b, and Dlgap2 for synaptic proteins.

Indeed, synaptic dysfunction is widely proposed as an initial insult leading to the neurodegeneration observed in AD [19, 21]. Although some of the protein phosphorylation changes observed were not related to cognition, use of this discovery-based approach represents a novel method for determining signaling events involved in specific memory processes. However, it should be noted that using stricter threshold to define differences in protein expressions will provide more solid evidence. In addition, morphology or functional analysis of these protein phosphorylation changes caused by LPS are also needed in our future studies.

Based on the results of protein-protein interaction analysis, Syngap1 is identified among the hub of synaptic plasticity. SynGAP is a protein abundant at the postsynaptic density of glutamatergic neurons and modulates synaptic strength by regulating the incorporation of AMPA receptors at the synapse [28]. Structurally, SynGAP is linked to postsynaptic scaffold proteins, which is critically involved in synapse density, synaptic physiology, and long-term potentiation [29]. In contrast, SynGAP disturbance has been linked to many neuropsychical diseases such as intellectual disability and autism spectrum disorders [30]. 1t has been shown that pSynGAP1 is required for AMPA receptor insertion and spine enlargement, whereas inhibition of SynGAP dispersion by CaMKII inhibitor prevents long- term potentiation [31]. This suggested that pSynGAP1 is regulated by pCaMKII and critically involved in synaptic plasticity. To test this hypothesis, we used a Cdk5 inhibitor roscovitine, which has been shown to upregulate pSynGAP1 level and improve cognitive impairment [13, 32, 33]. Consistently, we also showed that roscovitine reversed LPS-induced synaptic loss in the hippocampus. On the other hand, roscovitine is a Cdk5 inhibitor and is reported to have other effects such as anti-inflammatory property [34]. Thus, in addition to unregulated pSynGAP1, other mechanisms might also be involved in the beneficial effects of roscovitine for sepsis survivors.

Brain functions such as perception and cognition are based on particular functional network. It has been shown that neural assemblies involved in these cognitive functions will oscillate in a synchronized manner at specific frequencies while processing information [35]. In particular, theta oscillations are linked to various cognitive processes, especially for hippocampal-dependent memory function [36], whereas gamma oscillations are implicated in perception, learning, and memory [37]. To test whether altered oscillations in the brain are involved in LPS-induced long-term neurobehavioral abnormities, we used multichannel microwire array to record in vivo LFP, we found significantly decreased theta and gamma oscillations during locomotion in LPS-exposed animals, but did not significantly affect alpha and beta oscillation power. Although alpha and beta oscillations have also been involved in cognition [38], we found LPS selectively induced impairments in theta and gamma oscillations. Notably, we showed roscovitine rescued LPS-induced hippocampal oscillation disturbance and consequent neurobehavioral abnormities following LPS exposure, suggesting disturbance of pSynGAP1 is critical for hippocampal oscillation network impairment and may serve as a therapeutic target for sepsis related cognitive disorder.

In conclusion, our study provides new evidence that pSynGAP1 disturbance-mediated hippocampal oscillation network impairment might contribute to long-term neurobehavioral abnormities in sepsis survivors. However, further studies using more specific approach are needed to confirm our results. Moreover, other animal models of sepsis should also be used to confirm our current results.

## MATERIALS AND METHODS

### Animal model

The animal care and the experiment were approved by the Ethics Committee of First Affiliated Hospital of Zhengzhou University, Zhengzhou, China and were performed according to the Guide for the Care and Use of Laboratory Animals approved by the National Institutes of Health of the United States. One hundred seventy-two male C57BL/6 mice (3-4 months) were purchased from the Animal Center of Jinling Hospital, Nanjing, China. Due to animal death and different experimental purposes, we intentionally allocated more animals in the LPS or CLP group. Animals were randomly allocated to the following groups: control group (n =25), LPS group (n = 40), LPS + roscovitine group (n = 33), sham (n = 22) or CLP (n = 52) group. The mice were housed 4-5 per cage on a 12-h light–dark cycle in a room of 22-25 °C with food and water available *ad libitum*. Before the experimental study, animals were allowed to acclimatize for at least one week.

### Animal models of sepsis

We established the animal models of sepsis by utilizing LPS in a rodent model of sepsis as previously described [5, 6]. For LPS injection, mice received LPS (Escherichia coli endotoxin 0111: B4, Lot # 064M4125V, Sigma, Shanghai, China, 5 mg/kg). All the procedures were performed by an experienced investigator to keep the model stable.

CLP model was induced as we previously described [4]. Briefly, animals were anesthetized with 2% sodium pentobarbital (50 mg/kg; Sigma Chemical Co, St. Louis, MO, USA) by intraperitoneal (*i.p.*) injection and a 1-cm ventral midline laparotomy was performed. After then, the cecum was carefully exposed and ligated with a 4.0 silk suture, about 0.5 cm below the ileocecal valve. Subsequently, the cecum was perforated with 22–gauge needle and gently compressed to extrude a small amount of feces. Finally, the cecum was returned to the peritoneal cavity and the laparotomy was closed with 4.0 silk sutures. Immediately after the operation, animals received fluid resuscitation with normal saline solution (subcutaneously, 20 ml/kg of body weight) and antibiotic therapy (ertapenem, 20 mg kg^-1^; Merck Research Laboratory, USA). All mice were returned to their cages with free access to food and water. For sham group, animals were treated identically without ligation or puncture of the cecum.

### Drugs

Roscovitine (20 mg/kg, R-1234; LC Laboratories) or equal volume vehicle (0.2% dimethylsulfoxide) was injected *i.p.* daily for 3 days before and until the end of behavioral testing. The dosage of roscovitine was used based on previous study that 20 mg/kg/day roscovitine attenuated diabetes-related cognitive deficits [13].

### Behavioral experiments

Twelve months following LPS injection, a battery of well-established behavioral tests was used to assess behavioral alterations as we previously described [4, 39]. All behavioral studies were performed between 11:00 AM and 17:00 PM under dim lighting conditions. All the behavior of mice was recorded by a video camera (XR-XZ301, Shanghai Softmaze Information Technology Co. Ltd., Shanghai, China).

### Open field test

The exploratory activities of the mice were evaluated by the open field test. Each mouse was released in the center of the white plastic chamber (50 cm × 50 cm × 40 cm), and allowed to explore for 5 min. Total distance traveled and time spent in the center of the arena were automatically recorded by a video tracking system. At the end of testing, the arena was cleaned with 75% alcohol to avoid the presence of olfactory cues.

### Sucrose preference test

Anhedonia was measured by preference for a sucrose solution over water, using a two-bottle free choice method. Before test, mice were trained to consume two bottles of 1% sucrose solution for 24 h. On the testing day, each mouse was given two bottles of drinking containing either 1% sucrose solution or water for 24 h, where the position of the two bottles was switched to control for a side preference in drinking behavior at 12 h. Sucrose preference was calculated as sucrose consumption/(sucrose consumption + water consumption) × 100%).

### Fear conditioning test

The mouse was placed in the conditioning chamber (32 cm × 25 cm × 25 cm) for 3 min as an accommodation period and then one tone-foot-shock pairing (tone, 30 s, 65 dB, 1 kHz; foot-shock, 2 s, 0.8 mA) was delivered. Twenty-four hours later, mouse was placed back into the same chamber for 5 min without the tone and shock. The tone fear conditioning test was assessed 2 h after the contextual fear conditioning test in a novel chamber changed in the shape, color, and smell and the training tone was delivered for 3 min. The freezing behavior in these two chambers was video recorded, which was defined as the absence of all visible movement of the body except for respiration.

### Elevated plus maze test

Anxiety-like behavior was assessed by elevated plus maze test, where a central platform is connected to four arms (50 cm long, 10 cm wide, 70 cm above ground). Two opposite arms were enclosed by 20 cm high walls. Animals were placed onto the center platform with the head toward an open arm and allowed to move freely for 5 min. The sessions were videotaped by a camera over the center of the maze and the time spent in the open and closed arms. The maze was thoroughly cleaned with 70% ethanol between each test session.

### Forced swim test

This test measures depressive-like behavior with immobility taken as the dependent measure of behavioral despair. Mice were placed singly in a 4 litre clear plexiglass beaker (15 cm diameter 30 cm height) filled with water (20-24 °C) for 6 min, with the immobility scored in the final 4 min only. Time spent immobile (absence of movement except leg kicks to stay afloat) is then used as a measure of behavioral despair and helplessness.

### Electrophysiological recordings and analysis

Electrophysiological recordings and analysis were performed as we previously described [40]. Mice were anesthetized with 2% sodium pentobarbital in saline (40 mg/kg, *i.p.*; Sigma, St Louise, MO, USA) and fixed in a stereotaxic apparatus on a temperature-regulated heating pad set to maintain body temperature at 36–37 °C. After surgical preparation and craniotomies, local field potentials (LFP) were recorded from hippocampal CA1 region (2.1 mm posterior, 1.5 mm lateral, and 1.5mm depth) using a 8-channel microwire array. All electrodes were joined to a miniature connector and were then fixed to the skull using dental acrylic. After 7-day recovery period, LFP were recorded continuously (sampling rate = 1000 Hz; bandpass filter = 1–400 Hz) when the animals explored in the open arena. The recorded LFP were filtered by a 50 Hz notching filter to remove the powerline artifact. At the end of recordings, animals were deeply anesthetized and brains were removed and fixed for verification of electrode placement. All data analyses were performed by Neuroexplorer (Plexon Inc., Dallas, TX) software.

### Protein extraction, protein digestion, iTRAQ labeling, and MS/MS analysis

Protein extraction, protein digestion, iTRAQ labeling, and MS/MS analysis were described as previously [11, 12]. Briefly, the hippocampal tissues of 6 mice from each group were sacrificed by pentobarbital injection (50 mg/kg *i.p.*). Mouse tissues were quickly collected, rinsed with phosphate buffered saline (PBS) and flash frozen in liquid nitrogen. Samples were extracted and digested, and the tryptic peptides were labeled using the iTRAQ Reagent-8plex Multiplex Kit. The samples from the control group were labeled with iTRAQ tags 113 and 114, while tags 115 and 116 were used for the LPS group. iTRAQ labeling and tandem mass spectrometry analysis were carried out by Proteome Discoverer 2.1 (Thermo Fisher Scientific). The following parameters thresholds were set as : FDR ≤ 0.01, *P* value <0.05, and 1.2-fold change (expression difference >1.2-fold or <0.83-fold).

### Western blot analysis

Proteins extracted from the hippocampus were processed for western blot as we described previously [4]. In brief, an equal amount of protein (20 μg) was separated on 10% SDS-PAGE and transferred to PVDF membrane. Membranes were blocked with blocking buffer (5% BSA, 10 mM Tris pH, 7.5, 100 mM NaCl, and 0.1% tween-20) for 2 h. Membranes were then incubated overnight at 4 °C with primary antibodies rabbit anti-cleaved caspase-3 (1:1000; Cell Signaling Technology, USA), anti-cytochrome C (1:1000; Servicebio, Wuhan, China), anti-MAP2 (1:1000; Servicebio, Wuhan, China), anti-SynGAP1 (1:500; APExBIO, USA), anti-CamKII (1:1000; Abcam, Cambridge, UK), anti-pCamKII (1:1000; APExBIO, USA), anti-pSynGAP (Ser 1123, 1:500; bs-10392R, Beijing, China), and GAPDH (1:5000; Sigma St. Louis, MO, USA). After washing, membranes were incubated with respective HRP-conjugated secondary antibodies (1:5000; Santa Cruz Biotechnology, USA) for 2 h at room temperature. Protein bands were visualized and quantitated using ImageJ software (NIH Image, Bethesda, USA).

### Golgi staining

The brains of mice were assessed by a Golgi Stain Kit (#PK401, FD NeuroTechnologies, Columbia, MD, USA) at the ending of the behavioral tests as we previously described [41]. Briefly, mice were deeply anesthetized by sodium pentobarbital (60 mg/kg, *i.p.*; Sigma, St Louise, MO, USA) and rapidly sacrificed. The brains were immersed in impregnation solution (a mixture of solution A and B) and stored in the dark at room temperature for 3 weeks. Then, the brains were transferred into Solution C and stored for 7 days. Finally, the brains were sliced at a thickness of 100 μm, stained and then mounted on gelatin-coated slides. The dendrites from hippocampal neurons in CA1 region were captured with a confocal microscope (× 100 oil objective). Dendritic spine density were detected along CA1 secondary dendrites starting from their point of origin on the primary dendrite and the counting was performed by an experimenter blinded to the group of each sample.

### Statistical analysis

Statistical analysis was performed using GraphPad Prism 7.0 (GraphPad Software, La Jolla, CA, USA). Data are displayed as mean ± SEM. Comparisons between two groups were performed by independent-t test or Mann-Whitney U test where appropriate. Differences among multiple groups were assessed with one-way ANOVA followed by post-hoc Tukey multiple comparisons. The survival rate was estimated by Kaplan–Meier method and compared by the log–rank test. A *P* < 0.05 was considered statistically significant.
